# Author Correction: Electrical stimulation of smiling muscles reduces visual processing load and enhances happiness perception in neutral faces

**DOI:** 10.1038/s44271-025-00297-4

**Published:** 2025-07-22

**Authors:** J. Baker, HVV Ngo, T. N. Efthimiou, A. Elsenaar, M. Mehu, S. Korb

**Affiliations:** 1 Department of Psychology, University of Essex, Colchester, UK; 2 School of Psychology, University of Derby, Derby, UK; 3 Centre for Clinical Brain Sciences, University of Edinburgh, Edinburgh, UK; 4 ArtScience Interfaculty, Royal Academy of Art, Royal Conservatory, The Hague, The Netherlands; 5 Department of Psychology, Webster Vienna Private University, Vienna, Austria; 6 Department of Cognition, Emotion and Methods in Psychology, University of Vienna, Vienna, Austria

**Keywords:** Neuroscience, Psychology

Correction to: *Communications Psychology* 10.1038/s44271-025-00281-y, published online 02 July 2025

In the version of the article initially published, “N = 52” (number of study participants) should have read “N = 51” and has now been corrected in the Abstract and the legends for Figs. 3–5. In the Fig. 6 legend, “N = 52” has been corrected to “N = 48”. These corrections have been made to the HTML and PDF versions of the article.

