# Supplementary material for: Electrical stimulation of smiling muscles reduces visual processing load and enhances happiness perception in neutral faces
Source: Commun Psychol. 2025 Jul 2;3:94. doi: 10.1038/s44271-025-00281-y (PMC12222020; doi:10.1038/s44271-025-00281-y)
Supplement: Supplementary file 2 — Supplemental Information [file 44271_2025_281_MOESM2_ESM.pdf]

Supplementary Materials

PILOT STUDY 1: SELECTION OF STIMULUS MATERIALS 1

PILOT STUDY 2: TIMING OF SPONTANEOUS FACIAL MIMICRY 4

MAIN STUDY: ADDITIONAL RESULTS 8

Pilot study 1: Selection of stimulus materials

A first pilot study was carried out online to select stimulus faces to be used in the main study.

Participants

Twenty-one participants (9 women, mean age =35.9, SD = 5.65, range 28-55) were recruited through a mailing list at the University of Essex to an online study created in Psychopy3 (v3.2.4) for Windows (Peirce et al., 2019) and hosted using Pavlovia (pavlovia.org).

Stimuli

The stimulus set consisted of 22 avatars (11 female) generated using FaceGen (www.facegen.com). The avatars were created to have symmetrical faces with variance in the skin tone and face shape. All avatars were placed on a black background, with hair and ears removed. The generated faces were manipulated to express different emotions using FACSGen (Krumhuber et al., 2013; Roesch et al., 2011), which operates based on the facial action coding system (Ekman et al., 2002). Eleven images of each avatar were created using the following method. Firstly, three expressions were generated for each avatar (neutral, sad: AUs 1, 4, 15 at 100% intensity, AU7 at 80% intensity, and AU 11 at 60% intensity, and happy: AUs 6 and 12 at 100% intensity, and AU 7 at 25% intensity). This resulted in a neutral, 100% happy and 100% sad image for each avatar. Then, images were generated by morphing the neutral expression with the happy and sad expressions in 10% steps (from 10% to 50%), resulting in 11 images per avatar (i.e. 50% sad to 50% happy, through neutral at 0%). As such, 220 unique images were presented to participants.

Experimental procedure and task design

Participants were instructed to complete the task on a laptop or computer (i.e. not a mobile device). Each image was presented in the centre of the screen to the participants in a random order until a response was given. The absolute size of the image would vary depending on monitor specifications, however Pavlovia allows an auto-scaling feature which ensured that images occupied the same proportion of the screen for each participant (50% width, 65% height). Participants were required to rate the valance of each image on a visual analogue scale (from 0 to 10 in 100 points) by moving a red marker along a horizontal line with a computer mouse. The red marker was positioned at the centre position (5) at the beginning of each trial. The line was accompanied by three anchors; "Very sad" was positioned to the left of the line, "Neutral" was positioned below (middle), and "Very happy" to the right of the line. Participants were free to change the position of their response, and were informed to press the space bar to confirm.

Analysis

Prior to the selection of stimuli (detailed below), a two-step rejection procedure was used in order to remove extreme responses, and eventually to prune images from each intensity step. Firstly, the mean and SD was calculated for all ratings within each emotion step (i.e. all responses for images in the 50% sad category). Any ratings that were above or below two SD from the mean of the relevant category were removed (3.1% of all ratings). In the second step, the ratings of each unique image (e.g. 30% happy for avatar 4) were averaged across participants. Finally, the means and SD were calculated again for each intensity step, and images that were rated above or below 2 SDs from the mean of the respective intensity step were removed (10 images).

Selection of final stimulus set

Average ratings of all the remaining images were first sorted from lowest to highest. The top (happy) and bottom (sad) 10% of images (22 images each) were selected to be used in the main experiment. For the selection of images displaying neutral expressions, the middle 10% of images (21) images were selected. The resulting mean ratings for each emotion category were happy (7.19), sad (3.11), and neutral (4.98). Finally, ten avatars that had images within each of the emotion categories were selected for use in pilot 2 and the main study.

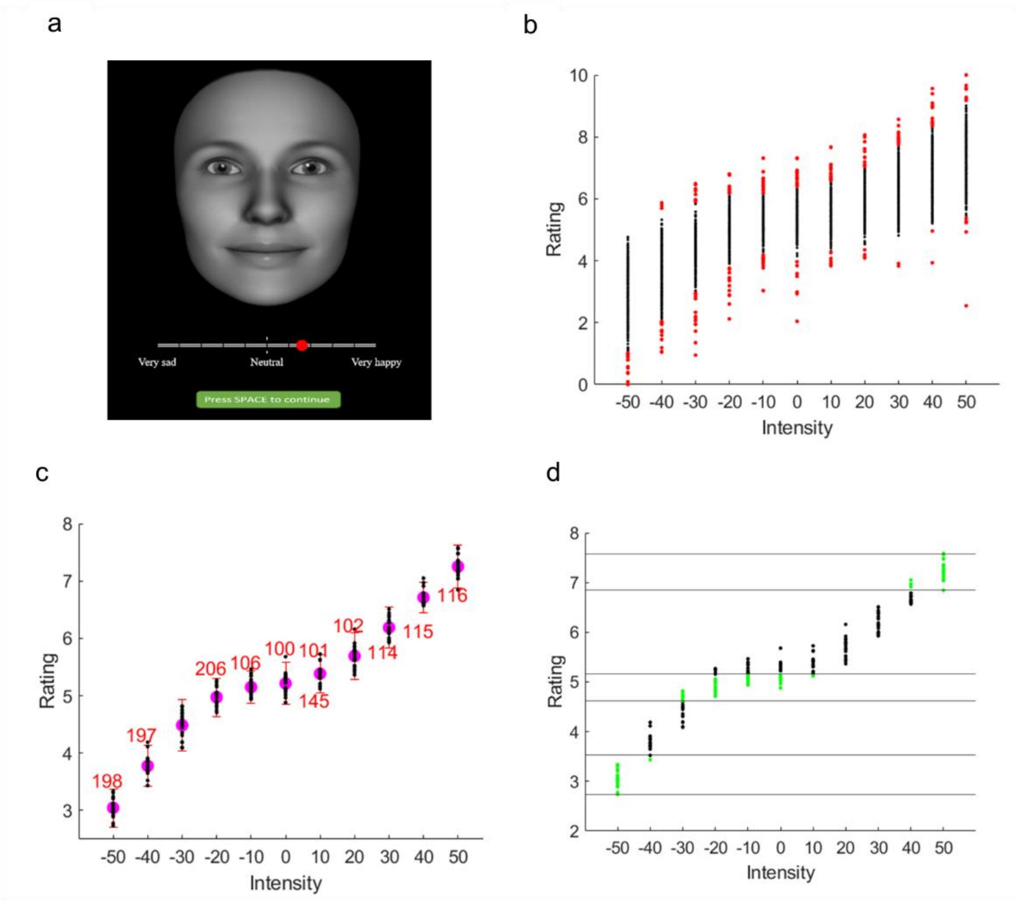

Figure S1. Pilot study 1. (a) An example trial in which participants rated the valance of a face by moving a red marker. (b) All provided ratings at each emotion intensity step ranging from left (sad, -50%) to right (happy, 50%). Red dots indicate ratings that were removed due to being more than 2 SD above or below from the mean ratings within the relevant intensity step. (c) Mean ratings (averaged across participants) for each unique image presented. Magenta dots show the mean rating for each intensity step. The red text indicates the image IDs that were removed due to being 2 SD away from the mean of the relevant intensity step. (d) Final ratings for each unique image. Horizontal lines show the top, middle, and bottom 10% of images (in green) that were selected to be used in subsequent experiments.

Pilot study 2: timing of spontaneous facial mimicry

A second pilot study had the objective to verify the time at which spontaneous facial mimicry would occur to our face stimuli. This would then inform when to apply fNMES in the late time window of the main study.

Participants

Twenty-two participants (16 women, mean age = 33, SD = 7.26, range 22-56) with normal or corrected to normal vision and with no facial hair were recruited for the study. Participants received £5 to take part. As a cover story, we informed participants that skin conductance (and not facial muscle activity) was being measured. Participants gave written informed consent before taking part. The study was approved by the local ethics committee (ETH2223-1598).

Stimuli

The stimulus set consisted of 20 images selected from the pool of images defined in pilot study 1. Ten avatars, each displaying neutral and happy expressions, were presented 10 times each, resulting in 200 trials in total (100 neutral and 100 happy facial expressions).

Experimental procedure and task design

Participants were sat in a comfortable chair roughly 60 cm away from a 23 inch PC monitor (width = 1920px, height = 1080px, 60 Hz) and were instructed to minimize any movement throughout the testing session. Following the signing of a consent form, the experimenter cleaned the skin of the participant (inside forearm and left cheek) with abrasive gel and an alcohol wipe so as to prepare for the application of the EMG electrodes and ground connection. A conductive gel was then placed into the electrode inlets of the shielded EMG sensors (EL254S by Biopac), which were then attached to the area above the ZM on the left

side of the face with double-sided adhesive rings. The ground electrode was placed onto the inside of the participant's upper forearm. Each trial began with a centrally presented fixation cross (horizontal = 1.19° and vertical = 1.19° of visual angle) for a jittered duration of 3500 to 4000 ms, which was then followed by a face stimulus (horizontal = 9.52° and vertical = 14.25° of visual angle) for 1000 ms presented at the centre of the screen. On 20% of trials, the phrase "Which emotion?" was presented following the face. In such cases, participants were required to press the left arrow key if the face they had just seen was presenting a neutral facial expressions, or to the press the right arrow key if it was presenting a happy expression. After 100 trials, participants were provided with an opportunity to take a break. Continuous EMG activity and behavioural responses were recorded throughout.

Facial EMG data acquisition and signal processing

EMG activity of the left ZM muscle was recorded continuously using a Biopac MP150 system with EMG100C amplifier (Biopac systems, UK) at 2 KHz with Acknowledge data acquisition software. EMG time series were analysed in Matlab (version R2019b, The Mathworks Inc.) partially using EEGlab (v2022.1) functions (Delorme & Makeig, 2004). Data were down-sampled to 1000 Hz and Cleanline (Mullen, 2012) was applied to reduce 50 Hz line noise. Data were then band-pass filtered between 20 and 200 Hz and rectified (taking the absolute value of each sample). Finally, an additional 40 Hz low-pass filter was applied and data were segmented to produce 1500 ms epochs (-500 ms pre-stimulus onset to 1000 ms following stimulus presentation). Noisy epochs (e.g. extremely large amplitudes) were removed based on visual inspection. To further remove bad trials, we implemented a rejection method based on peak and mean amplitudes both in the baseline period and main period of each trial separately. That is, for each emotion condition separately, we calculated the mean amplitude and mean peak amplitude in both the baseline and main period over all

trials in the respective condition. Individual trials were then removed if the peak and/or mean amplitude in the baseline period and/or in the main period exceeded 3 SDs from the condition mean. On average, 4.5% of neutral trials, and 6% of happy trials were removed per participant. Finally, epochs were baseline corrected by dividing each sample in a trial by the mean of the baseline period of that trial, thus resulting in values that express changes in EMG activation relative to baseline (%).

EMG analysis and results

We derived mean EMG activations per trial and participant in 10 time windows (0-100, 100-200 : 900-1000 ms) and analysed them with a 2 (emotion: neutral, happy) x 10 (time window) repeated-measures ANOVA in SPSS 29. Results revealed a significant main effect of emotion (F (1, 21) = 5.97, p =.023, η_p^2 = .222), whereby happy faces (M = 104.1, SD = 8.20) elicited greater ZM activation than neutral faces (M = 99.9, SD = 1.56) and a significant main effect of time (F (9, 21) = 4.14, p <.001, η_p^2 = .165). Most importantly, a significant emotion by time interaction was found (F (9, 189) = 4.52, p <.001, η_p^2 = .177, see figure S2). Post-hoc paired-sample t-tests revealed that the earliest time window in which EMG activity differed between neutral and happy trials was within the 500-600 ms window (p =.032), whereby happy faces (M = 106, SD = 11.63) resulted in larger EMG activations than neutral faces (M = 100.1, SD = 2.52). All proceeding time windows were also significant (600-700 ms: p =.042, 700-800 ms: p =.016, 800-900 ms: p =.041, 900-1000 ms: p =.027). As such, the findings of the present pilot study suggest the earliest onset of spontaneous facial mimicry to these specific happy facial expressions occurs only after 500 ms following the presentation of a face.

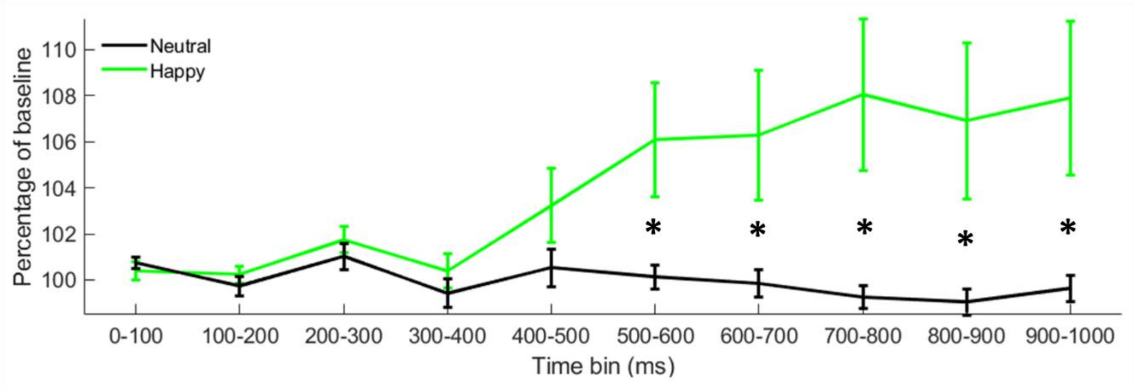

Figure S2. Zygomaticus major EMG activity (as % of baseline) in pilot study 2. EMG activity in response to viewing neutral (black) and happy (green) facial expressions. The earliest time bin in which significant differences were found between emotions was in the 500-600 ms time bin. Error bars show standard error. Asterisks indicate significance at the .05 level.

Main study: additional results

In addition to the models that identified a significant interaction between fNMES condition and N170 amplitude on choice, we also ran a model that predicted N170 amplitude by fNMES condition (off, early) and emotion (neutral, happy, sad): N170 ~ fNMES * emotion + (1 | participant), with off and neutral serving as the reference levels. This revealed a significant interaction between fNMES and happy faces ($\beta = 0.86$, $t = 4.66$, 95% *CI* (0.55, 1.17), $SE = 0.18$, $p < .001$). Posthoc tests revealed that in the off condition, N170 to happy faces ($M = 0.59$, $SE = 0.29$) was larger (more negative) than to both neutral faces ($M = 1.11$, $SE = 0.29$) [t (21871) = 4.65, $p < .001$] and sad faces ($M = 1.22$, $SE = 0.29$) [t (21871) = 5.14, $p < .001$]. In the early fNMES condition, however, N170 to happy faces ($M = 2.48$, $SE = 0.3$) was smaller (more positive) than to both neutral faces ($M = 2.14$, $SE = 0.29$) [t (21871) = 3.02, $p = .007$] and sad faces ($M = 2.14$, $SE = 0.29$) [t (21871) = 2.72, $p = .019$]. N170 to sad and neutral faces did not differ in either fNMES condition (all p > .05).

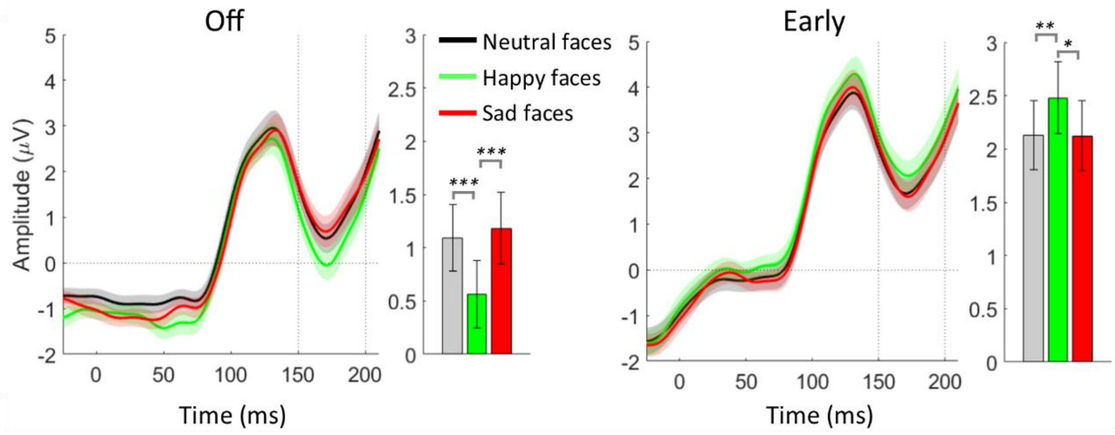

*Figure S3. N170 for happy and sad faces. N170 for happy (green) and sad (red) faces in the off (left) and early (right) fNMES conditions. In the off condition, N170 was larger (more negative) for happy than sad faces, however this was reversed when early fNMES was applied. Shaded areas and error bars show standard error. Vertical dotted lines represent time period from which the mean N170 amplitude was calculated. *** indicates p <.001, ** indicates p = .007, * indicates p = .019.*
