# Supplementary material for: Electrical stimulation of smiling muscles reduces visual processing load and enhances happiness perception in neutral faces
Source: Commun Psychol. 2025 Jul 2;3:94. doi: 10.1038/s44271-025-00281-y (PMC12222020; doi:10.1038/s44271-025-00281-y)
Supplement: Supplementary file 3 — reporting-summary [file 44271_2025_281_MOESM3_ESM.pdf]

nature portfolio

Corresponding author(s): Joshua Baker

Last updated by author(s): Apr 2, 2025

Reporting Summary

Nature Portfolio wishes to improve the reproducibility of the work that we publish. This form provides structure for consistency and transparency in reporting. For further information on Nature Portfolio policies, see our Editorial Policies and the Editorial Policy Checklist.

Statistics

For all statistical analyses, confirm that the following items are present in the figure legend, table legend, main text, or Methods section.

n/a	Confirmed	
☐	☒	The exact sample size (n) for each experimental group/condition, given as a discrete number and unit of measurement
☐	☒	A statement on whether measurements were taken from distinct samples or whether the same sample was measured repeatedly
☐	☒	The statistical test(s) used AND whether they are one- or two-sided<br>*Only common tests should be described solely by name; describe more complex techniques in the Methods section.*
☐	☒	A description of all covariates tested
☐	☒	A description of any assumptions or corrections, such as tests of normality and adjustment for multiple comparisons
☐	☒	A full description of the statistical parameters including central tendency (e.g. means) or other basic estimates (e.g. regression coefficient) AND variation (e.g. standard deviation) or associated estimates of uncertainty (e.g. confidence intervals)
☐	☒	For null hypothesis testing, the test statistic (e.g. F, t, r) with confidence intervals, effect sizes, degrees of freedom and P value noted<br>*Give P values as exact values whenever suitable.*
☐	☒	For Bayesian analysis, information on the choice of priors and Markov chain Monte Carlo settings
☐	☒	For hierarchical and complex designs, identification of the appropriate level for tests and full reporting of outcomes
☐	☒	Estimates of effect sizes (e.g. Cohen's d, Pearson's r), indicating how they were calculated

Our web collection on statistics for biologists contains articles on many of the points above.

Software and code

Policy information about availability of computer code

Data collection	EEG was collected using ANT Eego software (v. 1.9.2), behavioural data was collected using PsychoPy (v. 2021.1.4)
Data analysis	EEG Data were analysed in MATLAB (v. 2023b) using EEGlab toolbox (v.2020.0), with statistics run in R studio (v. 2023.12.1)

For manuscripts utilizing custom algorithms or software that are central to the research but not yet described in published literature, software must be made available to editors and reviewers. We strongly encourage code deposition in a community repository (e.g. GitHub). See the Nature Portfolio guidelines for submitting code & software for further information.

Data

Policy information about availability of data

All manuscripts must include a data availability statement. This statement should provide the following information, where applicable:

- Accession codes, unique identifiers, or web links for publicly available datasets
- A description of any restrictions on data availability
- For clinical datasets or third party data, please ensure that the statement adheres to our policy

We have provided a full account of our sample size determination, justifications for data exclusion, and comprehensive descriptions of all measures used within our research. The pre-registration, stimuli, tasks, analysis scripts, and data are openly accessible at Open Science Framework (https://osf.io/x2e8p/?view_only=122563b84a3f41c5950c9b03f46fe184).

Human research participants

Policy information about studies involving human research participants and Sex and Gender in Research.

Reporting on sex and gender	We collected self-reported sex for each participant, however we did not conduct any analyses on sex differences across our measured variables. Our study was not designed/powered to explore sex differences.
Population characteristics	See above
Recruitment	Participants were recruited through a number of channels (e.g. flyers, email lists, social media) and were financially compensated at a rate of £10 per hour
Ethics oversight	The study was approved by the University of Essex local ethics committee (ETH1920-0847)

Note that full information on the approval of the study protocol must also be provided in the manuscript.

Field-specific reporting

Please select the one below that is the best fit for your research. If you are not sure, read the appropriate sections before making your selection.

☐ Life sciences ☒ Behavioural & social sciences ☐ Ecological, evolutionary & environmental sciences

For a reference copy of the document with all sections, see nature.com/documents/nr-reporting-summary-flat.pdf

Behavioural & social sciences study design

All studies must disclose on these points even when the disclosure is negative.

Study description	This study explored the impact of facial neuromuscular electrical stimulation on emotion categorisation and EEG measures. Participants completed an experiment in which they labelled neutral, happy, and sad faces as either happy or sad, whilst receiving electrical stimulation to smiling muscles either during early visual processing, late visual processing, or not at all. As such, the study was entirely quantitative in nature.
Research sample	The participants were 51 adults (28 female, mean age = 22.9, SD = 3.65, range 18-33), with normal or corrected to normal vision, no current use of prescribed medication or history of illicit drug use, and no history of neurological or psychiatric illness. Participants were recruited from the University of Essex. We tested these individuals as these were the most readily available and most accessible for where we conducted the study. We believe our sample to be representative given the diversity of nationalities tested.
Sampling strategy	We have provided a full account of our sample size determination, justifications for data exclusion, and comprehensive descriptions of all measures used within our research in our preregistration. Specifically, We performed a power analysis for finding effects at the behavioural level (on neutral trials only) using simulation in R. In this analysis, our focus was on examining the impact of fNMES on categorising neutral facial expressions under two different timing conditions: early and late. Drawing from the study by Efthimiou et al. (2023), we assumed a small effect size, denoted as b = .09 for the early condition, indicating a 9% increase in the likelihood of classifying facial expressions as happy. For the late condition, we expected this effect to double in size, resulting in b = .18. We simulated a series of generalised linear mixed effects models using a binomial distribution and including a full random effects structure with all individual slopes and intercepts (response ~ fNMES + (fNMES | participant). Simulations were carried out with the simr package and ran 1000 simulations for five distinct sample sizes: 40, 45, 50, 55, and 60. The results from these simulations indicated that a sample size of 45 participants would provide us with an average statistical power of 87% (95% CI = 78.80% to 92.89%) to detect a significant effect of fNMES in the early condition.
Data collection	EEG data were collected using a 64 channel ANT eego sports system. Behavioural data were collected on a computer using a keyboard. Only the researcher and participant were present in the lab during testing. All trials were pseudo-randomised.
Timing	data were collected between November 2023 to March 2024
Data exclusions	The analyses concerning only neutral expressions (that is, comparisons between neutral faces labelled as happy with those labelled as sad), was carried out in 48 participants, as three participants had labelled all neutral faces as belonging to only one category.
Non-participation	No participants dropped out or decline to participate.
Randomization	Participants performed a series of randomised trials. The study was a within-subject design, and thus participants were not assigned to groups.

Reporting for specific materials, systems and methods

We require information from authors about some types of materials, experimental systems and methods used in many studies. Here, indicate whether each material, system or method listed is relevant to your study. If you are not sure if a list item applies to your research, read the appropriate section before selecting a response.

Materials & experimental systems

n/a	Involved in the study
☒	☐ Antibodies
☒	☐ Eukaryotic cell lines
☒	☐ Palaeontology and archaeology
☒	☐ Animals and other organisms
☒	☐ Clinical data
☒	☐ Dual use research of concern

Methods

n/a	Involved in the study
☒	☐ ChIP-seq
☒	☐ Flow cytometry
☒	☐ MRI-based neuroimaging
